# Human Hemorrhagic Pulmonary Leptospirosis: Pathological Findings and Pathophysiological Correlations

**DOI:** 10.1371/journal.pone.0071743

**Published:** 2013-08-12

**Authors:** Thales De Brito, Vera Demarchi Aiello, Luis Fernando Ferraz da Silva, Ana Maria Gonçalves da Silva, Wellington Luiz Ferreira da Silva, Jussara Bianchi Castelli, Antonio Carlos Seguro

**Affiliations:** 1 Institute of Tropical Medicine, São Paulo University Medical School, São Paulo, Brazil; 2 Department of Pathology, São Paulo University Medical School, São Paulo, Brazil; 3 Laboratory of Pathology, Heart Institute (InCor), São Paulo University Medical School, São Paulo, Brazil; 4 Laboratory for Medical Research, Nephrology Department, São Paulo University Medical School, São Paulo, Brazil; The University of Texas at San Antonio, United States of America

## Abstract

**Background:**

Leptospirosis is a re-emerging zoonosis with protean clinical manifestations. Recently, the importance of pulmonary hemorrhage as a lethal complication of this disease has been recognized. In the present study, five human necropsies of leptospirosis (Weil‘s syndrome) with extensive pulmonary manifestations were analysed, and the antibodies expressed in blood vessels and cells involved in ion and water transport were used, seeking to better understand the pathophysiology of the lung injury associated with this disease.

**Principal Findings:**

Prominent vascular damage was present in the lung microcirculation, with decreased CD34 and preserved aquaporin 1 expression. At the periphery and even inside the extensive areas of edema and intraalveolar hemorrhage, enlarged, apparently hypertrophic type I pneumocytes (PI) were detected and interpreted as a non-specific attempt of clearence of the intraalveolar fluid, in which ionic transport, particularly of sodium, plays a predominant role, as suggested by the apparently increased ENaC and aquaporin 5 expression. Connexin 43 was present in most pneumocytes, and in the cytoplasm of the more preserved endothelial cells. The number of type II pneumocytes (PII) was slightly decreased when compared to normal lungs and those of patients with septicemia from other causes, a fact that may contribute to the progressively low PI count, resulting in deficient restoration after damage to the alveolar epithelial integrity and, consequently, a poor outcome of the pulmonary edema and hemorrhage.

**Conclusions:**

Pathogenesis of lung injury in human leptospirosis was discussed, and the possibility of primary non-inflammatory vascular damage was considered, so far of undefinite etiopathogenesis, as the initial pathological manifestation of the disease.

## Introduction

Leptospirosis, a reemerging zoonosis, is an acute febrile illness occuring as large outbreaks throughout the world. It affects humans and/or animals in both urban and rural areas. The etiological agent is *Leptospira interrogans*, which can be transmitted from animal hosts to humans. Epidemiological and clinical aspects of the disease, as well as its pathogenesis and diagnostic methods, have been previously reviewed [1] [2] [3].

The most common and mildest form of clinical leptospirosis is anicteric, but an icterohemorrhagic presentation of the disease, known as Weil’s syndrome, can be found in 5–10% of all patients, leading to fatalities that typically arise from renal, cardiac and, more recently, from respiratory failure [3] [4].

Mild pulmonary involvement has been reported in 20–70% of leptospirosis patients, but this finding was often overshadowed by renal manifestations, which are now being succesfully controlled. Pulmonary hemorrhage, however, as cause of death in leptospirosis, has been the subject chiefly of epidemiological and clinical studies [4] [5] [6] [7] [8] and is now regarded as an important and frequent manifestation of the disease.

Septicemia of different etiologies, including leptospirosis, usually course with ionic dysfunction in the lung and kidney. Recently, specialized studies have focused on, the study of such alterations [9] [10] [11] [12] [13]. The aim of this work was to describe the main pathophysiological changes commonly seen in the lungs of leptospirotic patients, using antibodies expressed and detected by immunohistochemistry against vessels and cells involved in different electrolyte and water transport pathways, in an attempt to better understand the pulmonary failure in this disease.

## Methods

### 1- Human Samples

Five consecutive autopsy cases of patients with clinical and histological diagnosis of leptospirosis from a tertiary infectious diseases hospital were studied. The work received approval of the Ethics Committee from São Paulo University and all the necropsies were performed after written consent from the families or guardians, irrespective of the patients’ age, following the established rules from the University Hospitals. This includes the five patients whose lung fragments were used as controls.

The main clinico-epidemiological and laboratory data are presented in Table 1. Except for patient 2, who was 81 years of age, the average age was 29 years. The clinical and epidemiological information were in agreement with those observed in fatal leptospirosis (Weil’s syndrome). The average duration of illness was five days. The autopsies were complete, and tissue fragments were fixed in 10% neutral formalin, routinely embedded in paraffin, and stained with hematoxylin-eosin. All patients exhibited marked pulmonary involvement, as described in previous studies [14] [15] [16]. Besides macro and microscopic findings highly suggestive of leptospirosis, the immunohistochemical assay, as previously described [17], was positive mainly in the liver, and also in all the lung samples.

**Table 1:** Clinical data of leptospirosis patients.

Case number	Sex/Age (years)	Clinical and epidemiological information	Illness duration (days)
1	m/20	Fever, muscular pain, jaundice, acute renal failure, bipalpebral edema, pulmonary hemorrhage, epigastric pain and vomits. Low platelet count and leucocytosis.	4
2	f/81	Arterial hypertension, diabetes mellitus, muscular pain, jaundice, acute renal failure, acute respiratory failure. Patient refers contact with rats at home.	3
3	m/27	Fever, muscular pain, hepatomegaly, leukocytosis. Diffuse abdominal pain, vomits, massive pulmonary hemorrhage with hemoptisis. Acute renal failure. Positive serological tests for leptospirosis and B hepatitis. Previous contact with flood waters.	11
4	m/42	Jaundice, hepatomegaly, muscular pain, renal failure, acute respiratory failure. X rays showed micronodular interstitial infiltrate in both lungs. Leukocytosis.	4
5	m/27	Fever, muscular pain, jaundice, renal and pulmonary failure. Serological tests for leptospirosis, positive. Patient also had mansonic schistosomiasis	5

### 2- Immunohistochemical Assay

Immunohistochemistry to detect leptospiral antigen(s), pulmonary microvasculature and different electrolytes and water transport pathways was performed on paraffin sections using the antibodies listed in Table 2, in a standard protocol as previously described [17] [13]. Double immunohistochemical labelling and diaminobenzidine (DAB) visualization enhanced with nickel (DAB-Nickel) were also used in more representative slides. Immunohistochemical controls: Antibodies were tested in lung fragments of two non-leptospirotic patients dying of acute heart failure without definite macro and microscopic abnormalities, and also in three patients with terminal sepsis of different etiologies, and related lung pathology, usually represented by pulmonary edema and focal parenchymal hemorrhage. One patient, a sixteen-year-old male, had a lymphoproliferative disease and developed cavernous sinus thrombosis and terminal sepsis. The second patient was a 66-year-old female with cholelithiasis, relapsing acute and chronic cholecystitis, and sepsis. The third patient was a 52-year-old female with hepatocarcinoma, and who developed terminal sepsis after liver transplantation.

**Table 2:** Immunohistochemical protocols – essential data.

Primary antibody	Clone	Dilution	Link	Specificity
Anti-lepto	Polyclonal	1∶5.000	Envision System/AP DAKO	Leptospiral antigens
TTF1	Monoclonal	1∶500	Ultravision LP, Value Detection system,Lab Vision Corporation	Pneumocytes type II and Clara cells
CD34 cod. NCL-END	Monoclonal	1∶500	NOVOCASTRA cod. NCL - EWO	Glycoproteins of the endothelial cells membrane
Alpha ENaC Novus Biologicals cod NR p1 20097	Polyclonal	1∶700	Ultravision LP, Value Detection system,Lab Vision Corporation	Epithelial sodium channel
Anti-Connexin 43 C6219	Polyclonal	1∶400–1∶700	Ultravision LP,Value Detection System.Lab Vision Corporation	Gap Junction Protein
Aquaporin 1 ab 9566	Monoclonal	1∶1000	Ultravision LP,Value Detection System,Lab Vision Corporation	Water channels in humans endothelial cells
Aquaporin 1 ab 11023	Monoclonal	1∶20.000	Ultravision LP, Value Detection System,Lab Vision Corporation	Water channels in human endothelial cells
Aquaporin 5 ab78486	Polyclonal	1∶150	Ultravision LP, Value detection System,Lab Vision Corporation	Water channels in pneumocytes type I

### 3- Quantification

Morphometric analysis was performed as previously reported [18]. In summary, using a digital camera coupled to an optical microscope, we acquired 30 pictures of lung samples per case –15 from the main edema/hemorrage region and 15 from the peripheral area. The number of positive TTF-1 and AQP-5 cells was counted in each picture and corrected by the tissue area, measured using a 100-point grid (cells/tissue area), and expressed as cells/mm^2^. All the quantifications were performed using the software Image Pro Plus, Version 4.1 (MediaCybernetics, USA).

### 4- Confocal Laser Scanning Microscopy (CLSM)

Ten micrometer-thick paraffin sections of lung samples from one normal control and two leptospirotic cases, randomly selected, were applied to microscope slides and submitted to two-step immunofluorescence labelling. The slides were incubated with CD34 (dilution of 1∶250) and Aquaporin 1/Cod ab 9566 (dilution of 1∶300) primary monoclonal antibodies for 48 hours at room temperature following standard procedures [19]. The reactions were developed using secondary antibody conjugated with green fluorescent ***Alexa*** Fluor 488 (dilution of 1∶400), and the nuclei were counterstained with ***propidium iodide. T***he slides were kept in a dark chamber until observation at 20x and 40x objective magnifications, with water and oil immersion respectively, in a confocal laser microscope (model Zeiss LSM 510 META/UV), using LSM Image Examiner software (Carl Zeiss, Standort Göttingen, Germany) at the Confocal “Rede Premium” Multi-user Facility of the Heart Institute of São Paulo University.

## Results

Clinicoepidemiological data of the five patients were highly suggestive of leptospirosis. As expected in Weil’s syndrome, the illness was of short duration and this, associated with the usually delayed clinical diagnosis, contributed to the lack of important laboratory tests. However, the histopathological findings, and in particular, the immunohistochemistry, supported the diagnosis of leptosirosis by revealing tissue antigen deposits, mostly in the liver but also in all fragments of the lung.

Macroscopic pulmonary examination showed lungs with markedly increased weight. The cut surface revealed either nodular areas of hemorrhage, often confluent, or massive hemorrhage involving the lobes or even the entire lung parenchyma. A correlation between gross findings of the lung in human leptospirosis, essentially similar to ours, and the chest radiographs, was found by Marchiori et al., in their state-of-the-art review [16].

Histological findings showed septal congestion, multifocal alveolar hemorrhage and edema, occasionally with focal fibrin exudation. Macrophages were more numerous inside the alveolar lumina. The alveolar contour was visible inside the edematous and hemorrhagic regions, frequently enabling identification of the constituent cells. It is worth mentioning that in the peripheral, more preserved areas, the alveolar lining was made up of enlarged, apparently hypertrophic pneumocytes, occasionally in an arrangement resembling a glandular lining.

### 1- Immunohistochemistry

#### A- Leptospiral antigen(s) (LAg)

**Figure 1:**
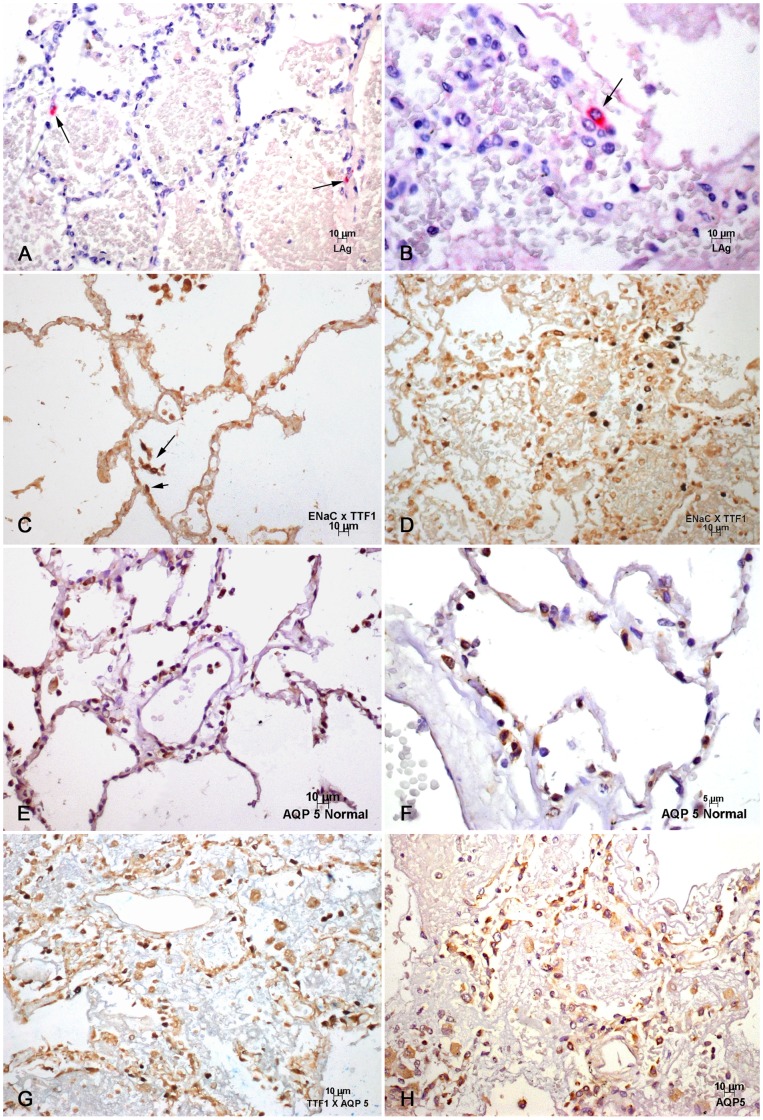
Immunohistochemical analysis of leptospirotic lungs: A and B: Antigenic leptospiral deposits (LAg) in cells of the human alveolar epithelium. The lumen is filled with plasma and red blood cells. Immunohistochemistry (IHC), alkalyne phosphatase. **C**: Normal human lung. Expression of ENaC in PI. Group of PII with nuclei marked by TTF1 (long arrow) is seen inside the alveolar lumen, close to the epithelial cell lining which exhibits few PII (short arrow). IHC, double labelling. **D**: Enlarged, possibly hypertrophic PI expressing ENaC made up mostly of the alveolar epithelium in leptospirosis. Groups of PII with nuclei expressing TTF1 are also part of the alveolar lining. IHC, double labelling. **E** and **F**: Normal human lung PI expressing aquaporin 5. The endothelial like shape of PI and the marked cytoplasmic expression of aquaporin 5 are present. IHC, DAB. **G** and **H**: Many enlarged, apparently hypertrophic PI expressing aquaporin 5 covers alveoli filled with plasma and red blood cells. PII are also present as part of the alveolar lining. IHC, 1G double labelling.

LAg were present in all cases, usually as small confluent dots, in the cytoplasm of few pneumocytes (Figures 1A and 1B), macrophages, and in rare cases, in the endothelial cells.

#### B- Epithelial cells

The TTF1 antibody was expressed in the nuclei in normal lungs in PII, which appeared as isolated groups of cells in their usual localization, in angles formed by the alveolar septa. In leptospirosis, pneumocytes expressing the TTF1 antibody were agreggated as small cellular groups or isolated cells, observed at the periphery of the hemorrhagic and edematous regions. It is notable that alveolar edema with septal widening was still frequently present, and that isolated pneumocytes expressing TTF1 could be observed inside and/or lining the alveolar spaces in the hemorrhagic and edematous areas. As expected, TTF1 nuclear expression was not present in the increased and occasionally hypertrophic macrophages scattered over the surface of alveoli, and sometimes percolating into the interstitium. Clusters of pigmented macrophages were also noted inside the air spaces. The quantitative analysis was performed in leptospirosis at the periphery of the microscopic slide, which showed either slight or absence of prominent edema and/or hemorrhage and at the central area where these findings were prominent. It showed a slight decrease in the number of TTF-1 positive cells, chiefly in leptospirosis, which was more severe when compared to the peripheral areas of sepsis and the controls (Graph 1).

Epithelial sodium channel (ENaC) expression was discrete in the cytoplasm of a few pneumocytes in normal lungs (Figure 1C). In leptospirosis, enhanced expression was detected in the cytoplasm and cell membrane of the cells of the alveolar lining, which were enlarged, and apparently more numerous, making the morphological distinction between pneumocytes difficult. This aspect was particularly apparent in the peripheral areas, where the cytological profile was usually more easily discernible. However, in the edematous and/or hemorrhagic areas, the outline of the alveolar lining could often be recognized, and ENaC expression was still apparently present in more preserved cells (Figure 1D).

The Aquaporin 5 expression in normal lung was found in the cytoplasm of PI, which showed a typical endotheliform appearance, lining the air spaces (Figures 1E and 1F). In leptospirosis PI were usually enlarged, apparently hypertrophic, chiefly at the periphery, but also frequently inside the edematous and/or hemorrhagic areas (Figures 1G and 1H). Quantitative analysis, as previously described for the TTF1 antibody, showed an increased number of aquaporin 5 positive cells in both leptospirosis and sepsis, but without significant difference from controls. The lack of statistical significance should take into account the limited number of cases included in this work (Graph 2).

#### C- Blood vessels (Figures 2A to 2H)

**Figure 2:**
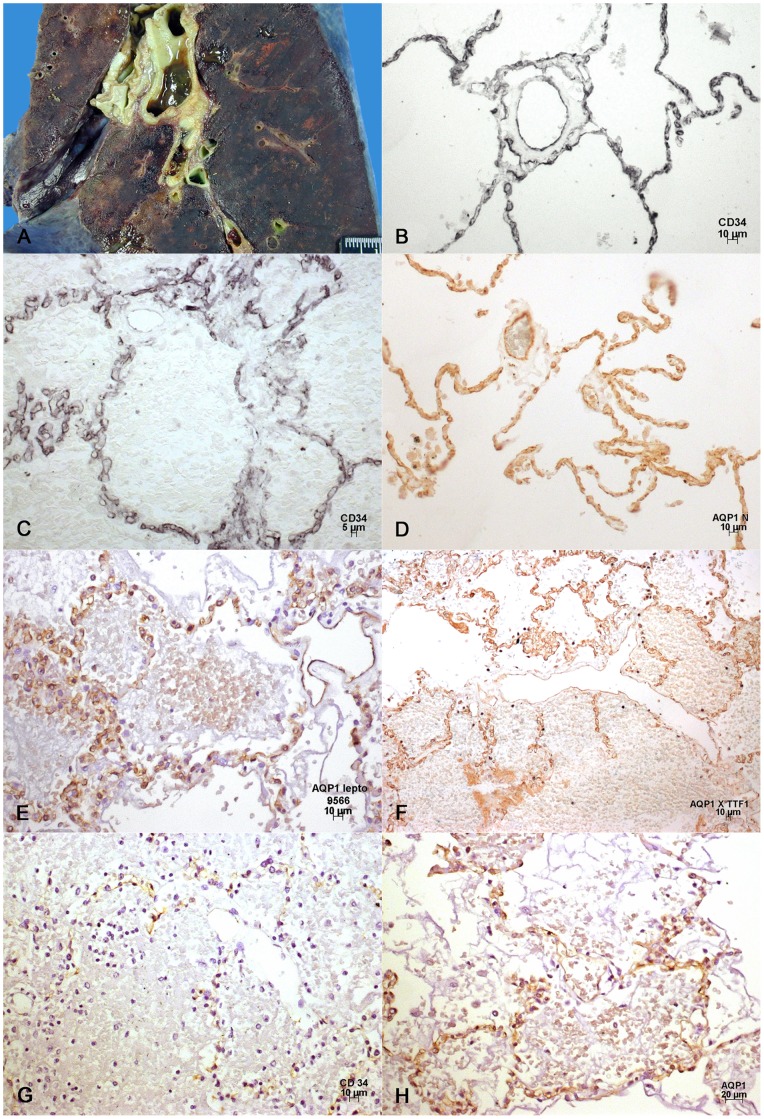
Gross feature and immunohistochemical analysis of microcirculation of leptospirotic lungs: A: Macroscopic aspect of the hemorrhagic pneumopathy in leptospirosis. Confluent hemorrhagic areas are present in the lung parenchyma. **B**: Microcirculation of the normal human lung. The capillary network is delineated in black, as well as the endothelium of a small branch of the pulmonary artery. IHC CD 34, DAB-Nickel. **C**: Human lung in leptospirosis. The capillary vessels are frequently dilated, with small gaps and areas of reduced and/or absent expression of CD 34. IHC CD 34, DAB-Nickel. **D**: Aquaporin 1 delineates the walls of the microcirculatory vessels in the normal human lung. It is also expressed in the endothelium of a small branch of the pulmonary artery. IHC, DAB. **E**: Aquaporin 1 expression is mostly preserved in areas of edema and apparent red blood cell deposits in human lung in leptospirosis. IHC Aquaporin 1, DAB. **F**: Capillaries of the pulmonary microcirculation express aquaporin 1 both at the more preserved periphery and inside the area of intraalveolar edema and apparent red blood cells extravasate. IHC Aquaporin 1. **G** and **H**: Both images were taken from similar regions of the slide. G shows CD34 reduced expression in areas of edema and hemorrhage and H the relative preservation of capillary expression of aquaporin 1. IHC, DAB.

**Figure 3:**
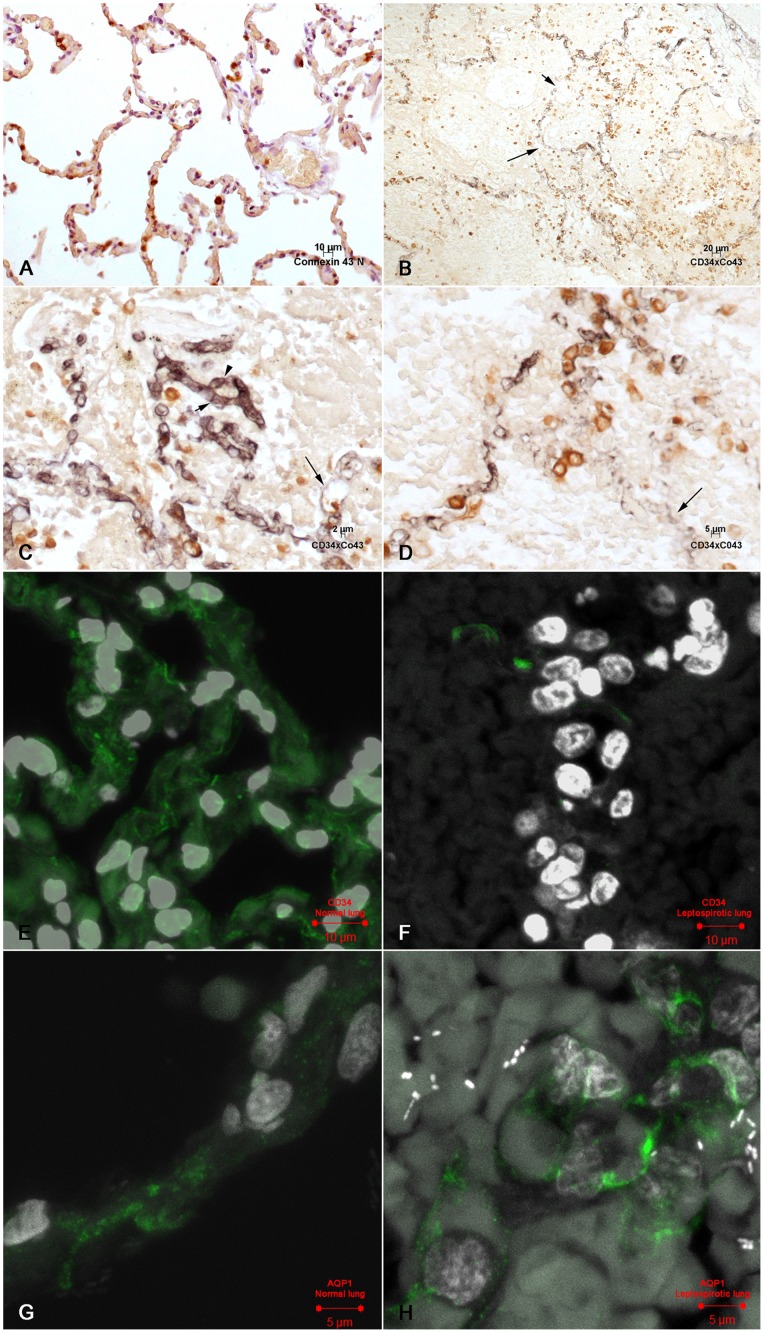
Immunohistochemical and confocal analysis of microcirculation of leptospirotic lungs: A: Connexin 43 is expressed in cells of the alveolar epithelial lining of a normal human lung. IHC, DAB. **B**: Lung in human leptospirosis. Connexin 43 is expressed both in pneumocytes of the epithelial lining and in those inside area of alveolar edema. Capillary with partial expression of CD34 in the endothelial membrane(long arrow) and a gap are present in the alveolar epithelium (short arrow). IHC, double labelling. **C**: Lung in human leptospirosis: Connexin 43 is expressed in the cytoplasm of isolated pneumocytes of the alveolar epithelial lining. CD34 is expressed in the capillary membranes. The short arrow and arrow head show apparently preserved capillary junctions. The long arrow points to a dilated capillary with reduced CD34 expression. Alveolar lumen is filled with plasma material and shadows of structures that might be interpreted as red blood cells. IHC, double labelling. **D**: Lung in human leptospirosis: Preserved expression of connexin 43 in groups and/or isolated pneumocytes. CD 34 shows an area with an almost complete lack of expression in the capillary membrane (long arrow) and irregular expression in the other capillaries. IHC, double labelling. **E**: Confocal microscopy of normal lung. The capillary walls are delineated by immunofluorescence. CD34 expression. **F**: Confocal microscopy in the human lung in leptospirosis: A few segments of capillary walls are delineated by immunofluorescence. CD34 expression. **G**: Confocal microscopy of normal human lung. Aquaporin 1 appears as fluorescent granules, probably in the cytoplasm of endothelial cells. **H**: Human lung in leptospirosis. Endothelial fluorescent granules are still present, probably in the cytoplasm of endothelial cells.

The CD34 antibody, due to its expression on the endothelial cell membrane, with or without enhancement by nickel, demonstrates the pulmonary alveolar microvasculature in normal lungs (Figure 2B). Expression was also observed in the endothelial lining of small branches of the pulmonary arteries. Similar expression was present with both aquaporin 1 antibodies, which in humans are able to detect endothelial cells (Figure 2D). In leptospirosis, the edematous and/or hemorrhagic areas showed dilated capillaries of the microvascular vasculature and extensive, but nevertheless focal areas with a partial or total lack of CD34 expression. Focally reduced CD34 expression was particularly visible when DAB–Nickel was used (Figure 2C). Gaps of different sizes were present, which might be interpreted either as sections of twisted dilated capillaries, or enlarged/disrupted endothelial cell junctions (Figures 3C and 3D). More preserved endothelial cells were prominent, occasionally with CD34 expression in the cytoplasm close to the cell nuclei and on the surface of the cell membrane facing the alveolar space. The CD34 expression was also either focally absent, or less expressed in more preserved and/or edematous areas at the periphery, but aquaporin 1 expression was still present and even apparently enhanced. Aquaporin 1 expression was also more preserved in the microvasculature of the alveolar spaces filled with hemorrhagic and edema fluid (Figure 2E and 2F), a finding that was less prominent as far as CD34 expression is concerned (Figures 2G and 2H). Expression of CD34 was partially absent in the endothelial lining of few small branches of the pulmonary arteries, a finding also seen less frequently with aquaporin 1. Overall, when compared with aquaporin 1, the damage to the microvascular bed of the lung appeared more severe when evaluated by CD34 expression.

Connexin 43 expression was present in most of the cells of the alveolar lining (Figure 3A). It was also detected in cells inside areas of edema and/or hemorrhage and even in the cytoplasm of more preserved endothelial cells. It is worth noting that the alveolar lining in leptospirosis may be discontinous in these regions, and isolated pneumocytes, or groups of pneumocytes expressing connexin 43, could be seen occupying part of the alveolar lumen (Figures 3B, 3C and 3D).

### 2- Confocal Microscopy

When compared with controls, confocal laser microscopy findings highlight a reduced endothelial membrane labelling of CD34, suggesting focal areas of discontinuity of the capillary wall (Figures 3E and 3F). Aquaporin 1 immunostaining was essentially preserved and apparently expressed inside the cytoplasm of endothelial cells, sometimes with a granular appearance (Figures 3G and 3H).

### 3- Control Cases

Cases of septicemia exhibited less marked but essentially similar findings of the lung in leptospirosis, except as far as the microcirculation is concerned. There were focal areas of edema and hemorrhage but these were less conspicuous when compared to cases of leptospirosis. However, close to these areas, groups of PI were also noted and transporters were similarly expressed. Also, macrophages were apparently more numerous than in the normal lung, but not as prominent as those observed in leptospirosis. The main differential finding was represented by the microvasculature, which was essentially similar to the normal lung except for a few small areas close to the edematous and/or small hemorrhagic areas, where foci of reduced and/or irregular CD34 expression appeared to be present.

## Discussion

Patterns of organ involvement and severity of leptospirosis are more recently evolving to frequent extensive lung damage [1] [4] [5]. The clinico-pathological finding that pulmonary hemorrhage can be a unique and often fatal manifestation of the disease received particular attention when in 1995, during the leptospirosis outbreak in Nicaragua, when pulmonary hemorrhage was the most frequent cause of death and, unlike classic icteric Weil’s disease, renal failure and jaundice were not present [4]. It is also important to emphasize that immunohistochemistry for the post-mortem diagnosis of human leptospirosis proved to be an extremely valuable and reliable tool during this particular outbreak of leptospirosis [4] [6]. Furthermore, immunohistochemistry to detect leptospirosis in horses also proved to be more sensitive and specific in tissue samples than serology using the microscopic agglutination test [20].

The alveolar epithelium, which covers almost the whole of the internal surface area of the lung, is composed of two cell types: squamous cells (pneumocytes type I – PI), which line 95% of the internal surface area of the lung, and granular or cuboidal cells (pneumocytes II – PII), which synthesize and secrete surfactant and cover the remaining 5% of the alveolus. PII are the progenitors of PI, which are incapable of cell division, and should proliferate after injury to restore alveolar epithelial integrity. Gas exchange takes place across the cytoplasm of PI, which incidentally also express aquaporin 5, a water channel that has high osmotic water cell membrane permeability [21]. PII contain ion channels, including the amiloride-sensitive epithelial Na^+^ channel (ENaC), Na^+^K^+^ ATPase and the cystic fibrosis transmembrane regulator [11] [22].

In pneumocytes, the Na-K-ATPase pump generates an osmotic driving force favorable to the entrance of sodium from alveolar lumen to the cell via ENaC channel situated at the lumen membrane of the pneumocyte. The osmotic gradient between the lumen and the interstitial space generated by sodium transport promotes the movement of water via the paracellular pathway. Water also crosses the cell via aquaporin 5 water channel [21]. An electroneutral cotransporter (NKCC1) at the interstitial membrane of the alveolar cells regulates the cellular volume. In endothelial pulmonary cells, another water channel (aquaporin-1) is responsible for water movement between the interstitium and the lumen of the vessels [33].

Alveolar epithelial cells also express gap junction proteins (connexins, Cx) involved in intercellular communication linking the cytoplasmic compartments of adjacent cells. Four connexins are expressed in cell culture, being Cx43 and Cx46 more abundant when compared with Cx 26 and Cx32 [23]. Cx43 was expressed also in the cytoplasm of preserved endothelial cells [23].

Leptospirosis may determine an acute lung injury that affects multiple components of the alveolocapillary membrane. Enhanced epithelial and endothelial permeability, the latter due to marked non-inflammatory circulatory damage, associated with impaired alveolar fluid clearance, induces prolonged respiratory failure and higher mortality. Alveolar fluid clearance results chiefly from the electroosmotic gradient created across the alveolar epithelium by active Na+ transport [24].

The lung in leptospirosis exhibits an alveolar cell non-specific reaction of PI, mainly at the periphery but also inside the large areas of intraalveolar edema and hemorrhage, with secondary focal disruption and occasional damage to the alveolar lining. PII are the progenitors for type I cells, but their decrease in number in leptospirosis, albeit slight, is probably associated with a compensatory enlargement, possibly hypertrophy, of PI that is visible at the periphery and even inside areas of lung edema and hemorrhage. We might speculate that the number of PI is probably linked to an early proliferative stimulus of PII in the initial stages of the lung damage, which progressively decreases when there is an unfavorable outcome of the disease. It is important to note that alveolar hyperplasia of PII was found in experimental models of septicemia, and endotoxin induction was considered in its pathogenesis [25].

For many years, it was accepted that only PII transported Na^+^ and Cl^-^ and that PI provided only a route for water absorption. Recent experimental and human physiopathological data [22] [26] presented evidence that PI contain functional epithelial Na^+^ channels (ENaC), as well as K^+^ channels and cystic fibrosis transmembrane regulator. Therefore, besides a high osmotic water permeability, attributable chiefly to its aquaporin 5 expression, PI also participate in active sodium transport, and this is what apparently is present in the lung in leptospirosis, as our immunohistochemical data regarding the ENaC detection seems to support.

Therefore, histopathological and immunohistochemical findings for leptospirosis showed what was expected in a non-specific attempt of alveolar edema clearance and the fundamental role of electrolytic and water transport by the epithelial alveolar lining. Furthermore, the preserved immunohistochemical findings of cells of the alveolar lining, including those in areas of edema and hemorrhage, suggests less pathophysiological damage than might be expected in such circumstances.

Connexin 43 expression seen in the epithelial cells inside areas of edema and hemorrhage might be interpreted as evidence of cytoplasmic communication between apparently preserved and/or less damaged cells, corroborating the above suggestion.

Leptospirosis can be regarded as a hemorrhagic septicemia, therefore the main findings involving vessels are essential in its pathogenesis. Discussion on the main pathogenetic mechanisms of the lung in leptospirosis involves either the presence of a toxin-mediated injury and/or an immune response of the host [3] [27]. However, in either of these possibilities, the microcirculatory role is predominant.

Damage to the pulmonary endothelium occurs without evidence of inflammation and/or disseminated intravascular coagulation in human leptospirosis. Furthermore, neither thrombocytopenia nor the decrease in clotting factors, which can occasionally be detected in leptospirosis patients, is sufficient to account for the bleeding diathesis observed [27] [28].

Nally et al. [29], in a guinea pig model of leptospirosis, found immunoglobulin and C3 deposited along the alveolar basement membrane in a similar pattern to that seen in Goodpasture syndrome. However, ultrastructural studies did not show the deposition of immunoglobulins in the capillary alveolar basal membrane, and histological examination of the kidneys did not demonstrate any pathological finding of Goodpasture disease. In any circumstance, the findings described suggested to the authors a possible role for an immune-mediated associated process.

Croda et al. [27] found fibrin deposits over the alveolar surface of human lungs in leptospirosis, and correlated these findings with necrosis of PI and PII, with cell leakage and hemorrhage into the alveolar lumen. They speculated that these might be the result of an initial increase in vascular permeability due to endothelial activation, which would permit leakage of immunoglobulins into the alveolar space, with further damage to the epithelial lining.

Lung tissue in patients with leptospirosis usually shows a much lower number of leptospires and antigen deposits, as detected by immunohistochemistry, when compared to liver and kidney tissue, suggesting that pulmonary abnormalities might be the result of leptospiral circulating products; so-called toxin(s). Leptospires and/or their antigen(s) appear to initiate cell injury by attaching to the cell membranes, a finding that is particularly visible in hepatocytes [16]. Leptospiral antigen was also detected by immunohistochemistry in the human lung on the luminal surface and cytoplasm of endothelial cells [28], a finding confirmed in the present work. The specific substance responsible for inducing this non inflammatory vascular injury remains unidentified, but possibilities include leptospiral outer membrane proteins, glycoproteins, hemolysins and lypopolysaccharides [15] [3]. Experimental data in guinea-pigs [30] and hamsters [31] also suggest vascular injury as playing a major role in the pathology of leptospirosis.

A recent work by Del Carlo Bernardi et al. [18], found, in vessels of human lungs in leptospirotic patients dying of hemorrhagic pneumopathy, an increased expression of intercellular adhesion molecule, vascular adhesion molecule, and Toll-like receptor, compared with the normal lung. Therefore, there is evidence that innate immune receptors and adhesion molecules participate in the pathogenesis of lung hemorrhage in leptospirosis.

Our findings are also in agreement with the main involvement of damaged microcirculation of the human lung in the pathogenesis of the pulmonary findings in leptospirosis. Leptospirosis exhibits well-known alterations of the endothelium in different tissues and organs, and it is attractive to suggest that the changed endothelial expression of CD34, and possibly aquaporin 1, as seen both by conventional and confocal microscopy, are part of a primary non-inflammatory injury to the microcirculation of the lung in leptospirosis. Altered expression of CD34, a heavily glycosylated type 1 transmembrane protein [32], suggests structural modifications of at least glycoproteins of the cell membrane, and possibly also of endothelial junctions, leading to alveolar edema and/or hemorrhage.

Aquaporin 1 is a water channel protein that is widely expressed in the human pulmonary vascular endothelium, particularly in endothelial cells of the vascular plexus around the airways, where it probably has a role in regulating the vascular permeability to water in the lung [33]. Its more preserved expression is notable, when compared to CD34 in the microvasculature of the edematous and/or hemorrhagic lung areas in human leptospirosis.

Aquaporin 1 is also naturally present in red blood cells and in many epithelial cells, where it has a major role in transcellular and transepithelial water movement. It is overexpressed in cells of certain histological types of human lung cancers, and it has been speculated that this finding is probably related to the need of the proliferating neoplastic cells to absorb water, using a minimal amount of energy [34]. A similar overexpression, increasing cell membrane water permeability, might be present in functionally more preserved endothelial cells in the lung in leptospirosis, accentuating the alveolar edema.

Macrophages are more numerous and hypertrophic in the human lung in leptospirosis, and are responsible, together with neutrophils, for the clearence of foreign bodies and microorganisms including leptospira and/or their products. Apparently, they do not have a major role in electrolytic or water transport. It is worth mentioning, however, that NKCC1, which is expressed in both epithelial and endothelial cells, is upregulated in the lung in leptospirosis, serving multiple functions ranging from ion transport, thus contributing to the pathology of pulmonary edema, to regulation of macrophage activation and antimicrobial activity [9] [10]. Additionally, PII may also act as immunoregulatory cells and together with macrophages, express Toll-like receptor 2, making them part of the innate immune defense mechanism [35].

The lungs of non leptospirotic patients dying of septicemia of different etiologies exhibited a predominance of focal, occasionally confluent areas of edema. Hemorrhage was usually less prominent when compared to leptospirosis. Marked vascular damage, as described in leptospirosis, was not present in the lungs of cases of septicemia. As a whole, non-leptospirotic septicemia exhibited milder, but similar findings as the ones found in leptospirosis.
