# Correction: Human Hemorrhagic Pulmonary Leptospirosis: Pathological Findings and Pathophysiological Correlations

**DOI:** 10.1371/annotation/af645b13-6dbb-4327-8e78-021ea552106a

**Published:** 2013-09-20

**Authors:** Thales De Brito, Vera Demarchi Aiello, Luis Fernando Ferraz da Silva, Ana Maria Gonçalves da Silva, Wellington Luiz Ferreira da Silva, Jussara Bianchi Castelli, Antonio Carlos Seguro

Graphics 1 and 2 were omitted from the article. They are available below:

Graphic 1: http://www.plosone.org/corrections/pone.0071743.g001.1.cn.tif

Graphic 2: http://www.plosone.org/corrections/pone.0071743.g002.1.cn.tif

